# Postural sway in young female artistic and acrobatic gymnasts according to training experience and anthropometric characteristics

**DOI:** 10.1186/s13102-021-00236-w

**Published:** 2021-02-12

**Authors:** Agnieszka Opala-Berdzik, Magdalena Głowacka, Grzegorz Juras

**Affiliations:** 1 Institute of Physiotherapy and Health Sciences, Department of Physiotherapy in Internal Diseases, The Jerzy Kukuczka Academy of Physical Education, 40-065 Katowice, Poland; 2 Institute of Sport Sciences, Department of Individual Sports, The Jerzy Kukuczka Academy of Physical Education, 40-065 Katowice, Poland; 3 Institute of Sport Sciences, Department of Human Motor Behavior, The Jerzy Kukuczka Academy of Physical Education, 40-065 Katowice, Poland

**Keywords:** Children, Gymnastics, Postural steadiness, Quiet standing

## Abstract

**Background:**

Gymnastics training enhances the development of postural control in children and adolescents. In competitive gymnastics, the training regimen is specific to the given gymnastic discipline and is usually followed from the early years of practicing. This study aimed to determine whether postural steadiness differed between young gymnasts practicing two distinct disciplines, and whether it was related to the duration of their training experience, age, and their anthropometric characteristics.

**Methods:**

Thirty 10–13-year-old females – ten artistic gymnasts, ten acrobatic gymnasts (training as “tops”), and ten non-athletes – were examined during 60-s quiet standing trials on a force platform with the eyes open and closed. Their postural sway was represented by directional components of centre of foot pressure mean velocity. Mann-Whitney U, Wilcoxon and Spearman’s ρ tests were used for analyses.

**Results:**

Anterior-posterior and medial-lateral centre of foot pressure mean velocities were not different between the artistic and acrobatic gymnasts (*p* > 0.05). In the artistic gymnasts, the duration of training experience, age, body height, body mass, and maturity offset were negatively correlated with the anterior-posterior centre of foot pressure mean velocity under eyes-open conditions. The acrobatic gymnasts’ body mass and BMI percentiles were negatively correlated with their anterior-posterior and medial-lateral centre of foot pressure mean velocities under both visual conditions (*r* ranged from − 0.64 to − 0.93; *p* < 0.05). The non-athletes’ centre of foot pressure mean velocities were non-significantly correlated with their age and anthropometric measures under both visual conditions (*p* > 0.05).

**Conclusions:**

The artistic gymnasts’ longer training experience, greater age, body height, body mass, and biological maturity were associated with better anterior-posterior postural steadiness when vision was available; the acrobatic gymnasts’ greater body mass and BMI percentiles were associated with better overall postural steadiness regardless of visual conditions. Relationships were observed between postural steadiness and discipline-specific training experience and anthropometric characteristics; however, causes and effects were not proven.

## Background

Gymnastics training experience begins in early childhood to achieve a high level of competitive success. It has been documented that practicing gymnastic disciplines since early years of life enhances the development of postural control [1–4]. In the postural control system, the central nervous system regulates information from visual, vestibular, and somatosensory sources and produces adequate motor responses to control body posture [5]. The neuromuscular control of natural standing can be examined in two planes. The dominant control in the sagittal plane is maintained by the ankle strategy, which provides the appropriate muscle tone of the ankle plantar and dorsiflexors. The control in the frontal plane is mainly maintained by the hip abductor and adductor muscle tone in a load/unload mechanism [6]. One of the methods used in the assessment of upright standing postural control is force-platform posturography. This method enables the analysis of centre of foot pressure (COP) oscillations which are related to postural sway. The basic assessment of postural sway concerns standing trials under normal and visual deprivation conditions. In healthy subjects, lesser postural sway is usually interpreted as better postural stability. Standing without visual cues challenges the postural control system and leads to increased postural sway; however, various human populations may use their redundant system differently to adapt to such conditions [5].

To date, developmental changes in the postural control system have been extensively investigated [7–12]. In adolescence, the improvement of the static and dynamic steady-state as well as proactive balance is observed compared to childhood. The maturation of the postural control system continues up to early adulthood, and balance performance might be differentially trainable during one’s youth [11].

A valuable insight into maturing postural control has been provided by studies comparing postural sway characteristics between children/young adolescent gymnasts and their non-athlete or other athlete peers under various laboratory conditions [1–3]. In young gymnasts, training experience results in superior control of standing on an unstable surface without visual information [3], as well as in better proprioceptive reweighting responses [1]. Increased gymnastic skills lead to less acceleration variability and body sway regularity and more stability during tandem stance, indicating a more automatic and more efficient postural control [13]. During stance perturbation, children/adolescent gymnasts demonstrated an increased muscle response latency of the upper body muscles located on the frontal aspect of the body compared to that of their non-athlete counterparts [14]. Interestingly, in quiet standing, superior postural steadiness was observed only in 5- to 7-year-old and not in 9- to 11- or 10- to 13-year-old gymnasts in comparison to age-matched groups of non-athletes [2, 15].

To the authors’ best knowledge, there have been no studies attempting to determine possible differences in postural control between gymnasts of different disciplines regardless of the age group. Although there are similarities in the training regimens of the gymnastic disciplines, there are also some distinctions between them. For example, female artistic gymnastics training leads to perfect performance of the routines on the vault, uneven bars, balance beam and floor. This discipline mainly requires phenomenal strength, power, flexibility, coordination and spatial awareness [16–21]. In turn, the effect of training in acrobatic gymnastics is the performance of the figures consisting of static and dynamic routines by the athletes working together. Within this discipline, the training regimens are role-specific. In the gymnasts who carry out supporting roles and are known as “bases,” among all gymnastic skills, their training emphasizes increasing strength and stability. The training of smaller and lighter “top” gymnasts focuses especially on displaying flexibility and agility [22–25]. In young adolescence, the duration and frequency of training sessions are higher in artistic compared to acrobatic gymnastics. The intensity of training for artistic gymnasts is the highest among the gymnastic disciplines [26, 27]. Therefore, the question arises as to whether discipline-specific gymnastics training which started in childhood translates into specific postural control characteristics of a simple daily activity such as quiet standing in young adolescence. It also appears that the length of training experience may be associated with postural sway characteristics. So far, it is also unknown whether the posturographic evaluation of quiet standing based on the traditional assessment of COP excursion is a sensitive enough method to distinguish the postural sway characteristics between young gymnasts practicing two disciplines.

Until now, little attention was given to the possible association between the anthropometric measures of children practicing gymnastics and their postural control. One study reported a poor correlation between body height and mass and the dynamic balance in pre- and postpubertal female gymnasts practicing trampoline [3] and the other study demonstrated a moderate to strong negative correlation between the body mass of 10- to 13-year-old female acrobatic gymnasts and their postural sway velocity, suggesting better postural steadiness in the heavier athletes [15]. In 6- to 8-year-old artistic gymnasts, simple balance tests indicated negative association of body height with static balance, and positive association with dynamic balance. The opposite phenomenon was observed in regard to body mass [28]. The gymnasts’ anthropometric measures have been closely investigated in relation to their specific skills [16, 24, 25]; however, the investigation of their relationship with quiet standing postural control may provide important additional information. If such a relationship exists, the findings might have a practical application in selecting children for certain gymnastic disciplines. Gymnastic performance is also related to their biological maturity [29], therefore its relationship with postural sway should be investigated as well.

The primary aim of the study was to determine whether postural steadiness (reflected by quiet standing postural sway velocities) would be different between young adolescent artistic gymnasts and acrobatic (having the role of the “top”) gymnasts under normal and visual deprivation conditions. The secondary aim was to investigate whether the duration of gymnasts’ training experience, their age, maturity offset, and anthropometric measures would be associated with their postural steadiness. To facilitate the proper interpretation of correlation analyses in the gymnasts, the aim was also to explore the association of age, maturity offset, and anthropometric measures with postural steadiness in the age-matched non-athletes. We hypothesized that the postural sway characteristics might be linked to discipline-specific training and anthropometric characteristics.

## Methods

### Participants

Thirty 10–13-year-old females (ten artistic, ten acrobatic gymnasts, and ten non-athletes) participated in the study. The gymnasts were recruited from training centres and the non-athletes were recruited from an elementary school in Upper Silesia, Poland. The inclusion criteria for the gymnasts of both groups were being at the national performance level and practicing for at least 7 h per week. The inclusion criteria for the acrobatic gymnasts were carrying out the role of the “tops” (“middles” and “bases” were excluded from the study because of different anthropometric characteristics, training regimens and usually older age at the national performance level). The inclusion criteria for the non-athlete females were no experience in practicing sports and participation only in general physical education classes at the elementary school (3 h per week). Exclusion criteria were uncorrectable vision disorders, overweight, greater body height than the average height for their age, and any neurological or musculoskeletal abnormalities that could affect postural stability. The aim of the study and experimental procedures were explained to all study participants and their legal guardians, and written informed consent was obtained from the participants and the guardians. The gymnastics training centres provided the data regarding the athletes’ training frequency and the duration of the training period. The artistic gymnasts practiced 5 times per week for 2.5 h, and most (seven of ten) began their training experience at the age of 6 years. The acrobatic gymnasts practiced 3–5 times per week for 2–2.5 h. Of ten acrobatic gymnasts, eight began practicing at the age of 8 years. The characteristics of the participants are presented in Table 1.

**Table 1:** Characteristics of national-level female artistic gymnasts, acrobatic gymnasts (training as “tops”), and non-athletes^a^

	ARG (***n*** = 10)	ACG (***n*** = 10)	Non-athletes (***n*** = 10)
**Age (years)**	11.1 ± 1.29; 10–13	11.3 ± 0.67; 10–12	10.8 ± 0.79; 10–12
Body height (cm)*	139.8 ± 5.85; 132–150	132.5 ± 5.23; 124–140	146.5 ± 6.43; 136–155
Body mass (kg)**	29.6 ± 3.6; 24–35.5	26.9 ± 4.31; 20–34	36.2 ± 4.82; 29–41.5
BMI percentile***	13.2 ± 9.0; 3–27	17.3 ± 16.69; 0–49	39 ± 24.14; 9–84
**Maturity offset**	−1.1 ± 1.00; −2.1-0.5	− 1.4 ± 0.52; (− 2.3) - (− 0.8)	−1 ± 0.75; − 2-0.1
Gymnastics experience (years)****	4.8 ± 0.92; 4–6	3.3 ± 0.67; 2–4	0
Training frequency (hours/week)*****	12.5 ± 0; 12.5	9.1 ± 1.45; 7–10	0

*ARG* artistic gymnasts, *ACG* acrobatic gymnasts

^a^ Data are presented as means ± standard deviations; ranges

*ARG vs ACG: *p* = 0.01, *r*_*ES*_ = 0.54; ARG vs non-athletes: *p* = 0.02, *r*_*ES*_ = 0.5; ACG vs non-athletes: *p* = 0.00004, *r*_*ES*_ = 0.81. **ARG vs non-athletes: *p* = 0.007, *r*_*ES*_ = 0.59; ACG vs non-athletes: *p* = 0.0007, *r*_*ES*_ = 0.7. ***ARG vs non-athletes: *p* = 0.001, *r*_*ES*_ = 0.69; ACG vs non-athletes: *p* = 0.04, *r*_*ES*_ = 0.46. ****ARG vs ACG: *p* = 0.002, *r*_*ES*_ = 0.72. *****ARG vs ACG: *p* = 0.00001, *r*_*ES*_ = 0.93 (Mann-Whitney U test)

*ARG* artistic gymnasts, *ACG* acrobatic gymnasts, *BMI* Body mass index, Maturity offset - years from age at peak height velocity.

### Procedure

The recording of the subjects’ body height and mass preceded the postural steadiness tests. Based on height, mass and age, individual female BMI percentile rankings were calculated according to the international BMI cut-off points for children established by the International Obesity Task Force (Table 1). To estimate biological maturity, the following maturity offset regression equation for girls was used:

$$ \boldsymbol{Maturity}\ \boldsymbol{offset}=-\mathbf{7.709133}+\left(\mathbf{0.0042232}\times \left(\boldsymbol{age}\times \boldsymbol{height}\right)\right) $$

The result of this equation indicates the number of years from the age at peak height velocity [27] (Table 1).

To assess postural sway, the subjects were instructed to stand barefoot, as still as possible (the arms relaxed by the sides), with the feet approximately hip-width apart on a stable force platform (AMTI AccuGait, Watertown, MA, USA). Before the measurement, they performed one introductory trial. Four 60-s quiet standing trials were conducted: two with the eyes open (looking straight ahead at a wall 3 m away) and two with the eyes closed [29]. After each trial, the subjects stepped off the platform and rested up to 1 min to avoid any discomfort. The COP signals transmitted from the force plate were amplified and sampled at a frequency of 100 Hz. They were filtered with a 4th order low pass Butterworth filter at a 7-Hz cut-off frequency. The COP mean velocity was chosen for analysis because, according to the systematic review of test-retest reliability studies concerning bipedal static posturography, this parameter shows consistently acceptable reliability values and it is the most reliable among the traditional COP parameters [30]. The COP mean velocity showed an excellent reliability in the assessment of children and young adolescents (6–14 years) [31]. Moreover, in our preliminary Factor Analysis of the traditional COP parameters the COP mean velocity had the highest absolute value of factor loading. This measure is defined as the average speed of COP movement over the time of a trial. The directional (anterior-posterior and medial-lateral) subcomponents of the COP mean velocity were normalized to the subject height because of significant between-group differences concerning this factor [32]. The subcomponents were computed on the basis of the means of two trials for the eyes-open conditions and two trials for the eyes-closed conditions [31].

### Statistical analyses

Statistical analyses were performed on the directional subcomponents of COP mean velocities (eyes-open and eyes-closed conditions), training experience, age and anthropometric characteristics. The age and anthropometric factors of the gymnasts and non-athletes were compared with the use of the Mann–Whitney U test. This test was also used to compare the training experience and COP measures between the artistic and acrobatic gymnasts. Wilcoxon test was applied to compare eyes-open with eyes-closed trials in each group of gymnasts. The results of the comparison analyses concerning gymnasts and non-athletes coming from the same research project have been reported [15]. Effect size (ES) for nonparametric data was calculated (r = Z/√N)**.** The following thresholds were used to interpret ES: r > 0.10 small; r > 0.30 medium; and r > 0.50 large [33]. Spearman’s Rank Correlation Coefficient was used to test whether the directional components of COP mean velocities were correlated with training experience and anthropometric characteristics in gymnasts and with anthropometric characteristics in non-athletes. A conventional approach to interpreting the absolute magnitude of the correlation coefficient was used: 0.00–0.10 negligible; 0.10–0.39 weak; 0.40–0.69 moderate; 0.70–0.89 strong; and 0.90–1.00 very strong correlation [34]. Additionally, training experience, age, and anthropometric measures were correlated in each group of gymnasts. These between-factor correlation analyses facilitated the determination of the dynamics of body maturation in relation to training experience and age. The results allowed for a better understanding and interpretation of the correlations between the COP velocities of the gymnasts and all of the above factors. The level of significance was set to *α* = 0.05. The analyses were performed using Statistica v.13 (StatSoft Inc., Tulsa, OK, USA).

## Results

### Between-group and between-condition comparisons

**Fig. 1:**
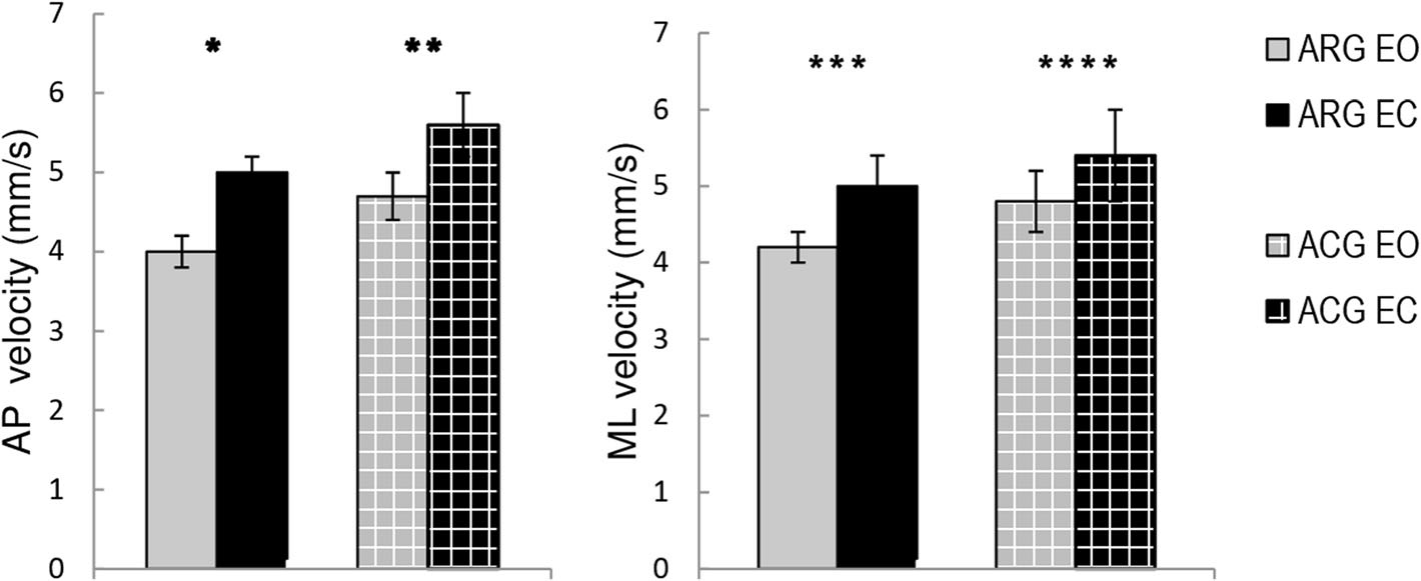
Normalized to body height centre of pressure mean velocities during 60 s of quiet standing in artistic (*n* = 10) and acrobatic (*n* = 10) gymnasts. Data shown as the means ± standard errors. Between group differences (Mann-Whitney U): *p* > 0.05; Between-session differences - EO vs. EC (Wilcoxon test): *p = 0.005, *r*_*ES*_ = 0.63; **p = 0.009, *r*_*ES*_ = 0.58; ***p = 0.04, *r*_*ES*_ = 0.47; ****p = 0.047, *r*_*ES*_ = 0.44. ARG – artistic gymnasts, ACG – acrobatic gymnasts; EO - eyes open, EC – eyes closed; AP – anterior-posterior plane, ML – medial-lateral plane.; *r*_*ES*_ – effect size

There were nonsignificant differences between the COP mean velocities of the young adolescent female artistic and acrobatic gymnasts (Fig. 1). In the between-group comparison of eyes-open trials, the effect size for the anterior-posterior COP mean velocity was medium (*r*_ES_ = 0.32) and for medial-lateral COP mean velocity it was small (*r*_ES_ = 0.17). In the comparison of eyes-closed trials, the effect size for both anterior-posterior and medial-lateral planes was small (*r*_ES_ = 0.22 and 0.09, respectively). The COP mean velocities were increased during the trials with eyes closed compared to the eyes-open trials in each of the two groups. In both artistic and acrobatic gymnasts, the effect size between eyes-open and eyes-closed trials was large for the anterior-posterior plane and medium for the medial-lateral plane (Fig. 1).

Although the level of performance of all the gymnasts was national, the duration of the artistic gymnasts’ training experience was significantly greater. Additionally, the artistic gymnasts practiced more hours per week compared to the acrobatic gymnasts (Table 1). There was a nonsignificant difference and small effect size in the age, body mass, BMI percentile, and maturity offset of the gymnasts (*p* > 0.05, *r*_ES_ = 0.14, 0.28, 0.01, and 0.10, respectively); however, the artistic gymnasts’ body height was significantly greater than that of the acrobatic gymnasts. The non-athletes' body height, mass and BMI percentile were significantly greater than that of the gymnasts (Table 1).

### Correlations

#### Correlations between COP mean velocity and the duration of the gymnastics experience, age, body height, and maturity offset

**Fig. 2:**
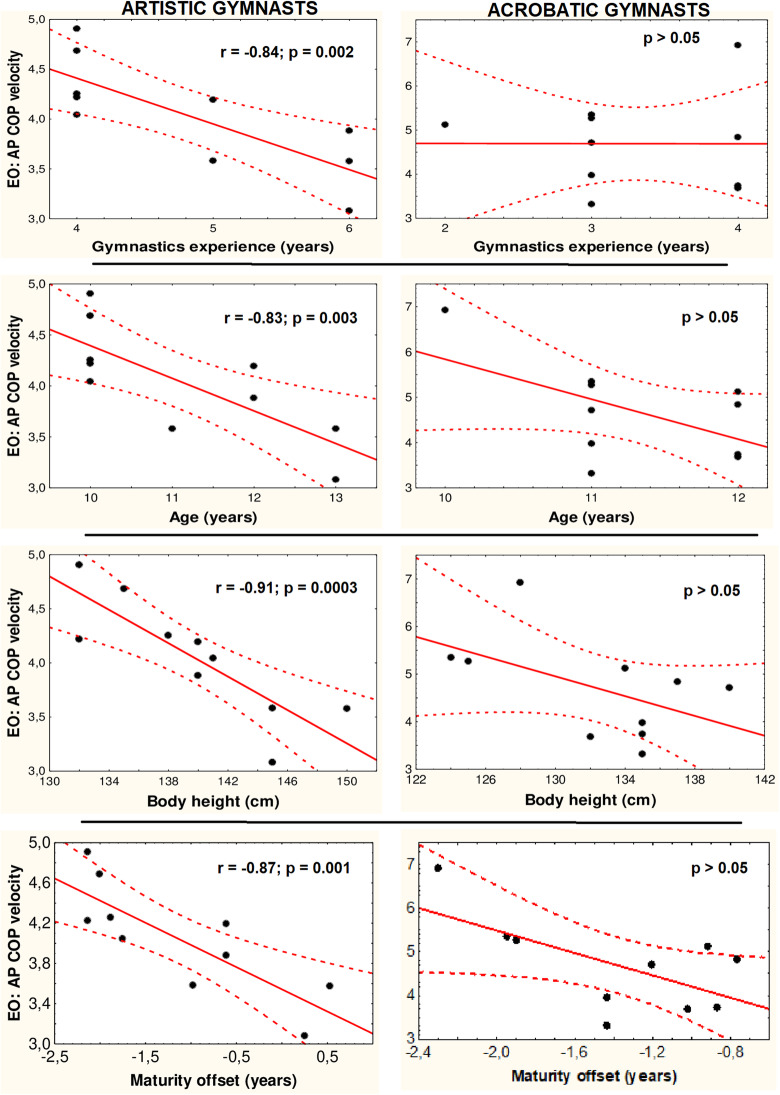
Spearman’s correlations of young adolescent female artistic and acrobatic gymnasts’ normalized to body height anterior-posterior centre of pressure (AP COP) mean velocities acquired during 60-s quiet standing eyes-open (EO) trials with the durations of their training experience, ages, body heights, and maturity offsets (correlations for medial-lateral plane and both planes in eyes-closed trials are not shown; *p* > 0.05 in both groups). Maturity offset - years from age at peak height velocity

Eyes-open trials: the results of the correlation tests in the artistic and acrobatic gymnasts between their anterior-posterior COP mean velocities and their training experience, their ages, body heights, and maturity offsets are shown in Fig. 2. In both groups, nonsignificant correlations were observed between the medial-lateral COP mean velocities and these measures (*p* > 0.05). Eyes-closed trials: the directional components of the COP mean velocities in the artistic or acrobatic gymnasts were non-significantly correlated with the duration of their training experience, their ages, and their body heights (*p* > 0.05). In the non-athletes, the directional components of the COP mean velocities were not significantly correlated with their ages, body heights, and maturity offsets under both visual conditions (*p* > 0.05).

#### Correlation between COP mean velocity and body mass

**Fig. 3:**
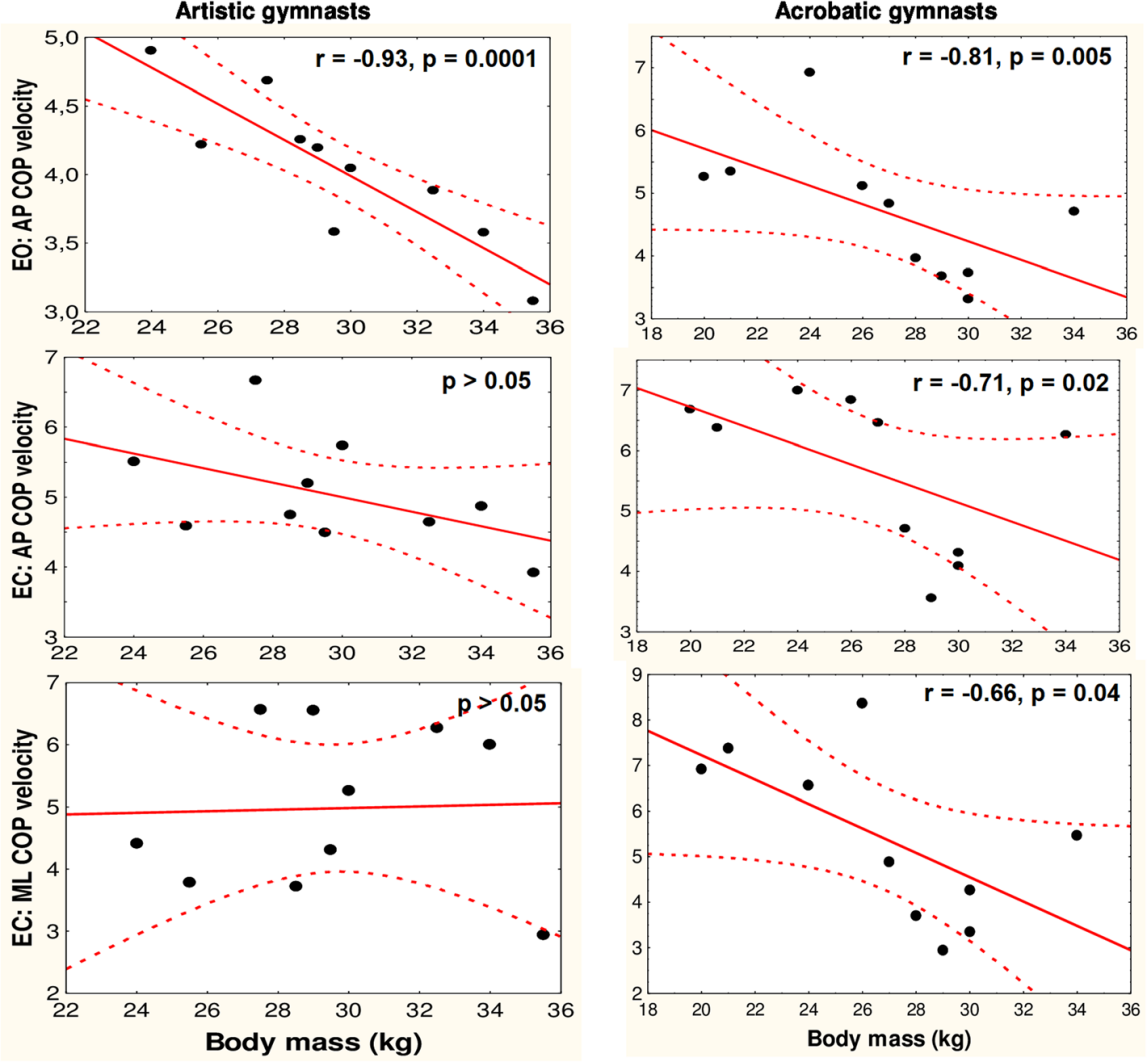
Spearman’s correlations of young adolescent female artistic and acrobatic gymnasts’ normalized to body height centre of pressure (COP) mean velocities acquired during 60-s quiet standing trials with their body masses (correlations for medial-lateral plane in eyes open trials are not shown; *p* > 0.05 in both groups). EO – eyes open, EC – eyes closed trial; AP – anterior-posterior, ML – medial-lateral plane

Eyes-open trials: the results of the correlation tests in the artistic and acrobatic gymnasts between their anterior-posterior COP mean velocities and their body masses are shown in Fig. 3. In both groups, nonsignificant correlations were observed between the medial-lateral COP mean velocities and body masses (*p* > 0.05). Eyes-closed trials: the results of the correlation tests in both groups of gymnasts between the directional components of the COP mean velocities and their body masses are shown in Fig. 3. The non-athletes’ directional components of the COP mean velocities were not significantly correlated with their body masses under both visual conditions (*p* > 0.05).

#### Correlation between COP mean velocity and BMI percentile

**Fig. 4:**
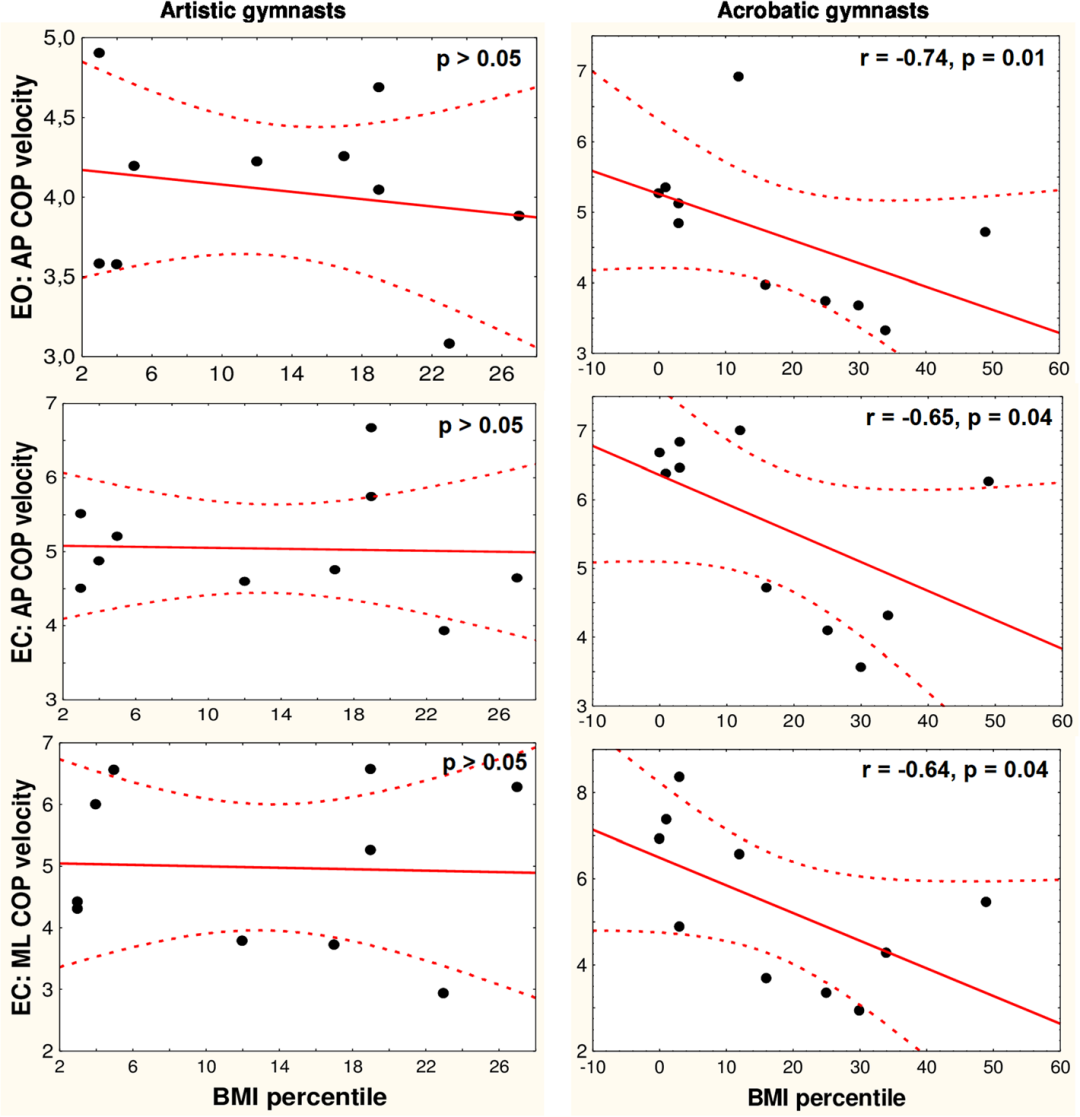
Spearman’s correlations of young adolescent female artistic and acrobatic gymnasts’ normalized to body height centre of pressure (COP) mean velocities acquired during 60-s quiet standing trials with their BMI percentiles (correlations for medial-lateral plane in eyes open trials are not shown; *p* > 0.05 in both groups). EO – eyes open, EC – eyes closed trial; AP – anterior-posterior, ML – medial-lateral plane

Eyes-open trials: the results of the correlation tests in the artistic and acrobatic gymnasts between their anterior-posterior COP mean velocities and their BMI percentiles are shown in Fig. 4. In both groups, nonsignificant correlations were observed between the medial-lateral COP mean velocities and BMI percentiles (*p* > 0.05). Eyes-closed trials: the results of the correlation tests in both groups of gymnasts between the directional components of the COP mean velocities and their BMI percentiles are shown in Fig. 4. The non-athletes’ directional components of the COP mean velocities were not significantly correlated with their BMI percentiles under both visual conditions (*p* > 0.05).

#### Additional between-factor correlations

The strengths of the correlations between training experience, age, and anthropometric measures were determined to facilitate the proper interpretation of the main results concerning correlations between the COP velocities of the gymnasts and the above factors. Significant between-factor correlations are presented in Table 2.

**Table 2:** Significant ^a^ Spearman’s ranks correlation coefficients between training experience, ages, and anthropometric measures in young adolescent female artistic gymnasts and acrobatic gymnasts (training as “tops”)

**ARTISTIC GYMNASTS (*n*** **= 10)**						
**Measure**	**Gymnastics experience**	**Age**	**Body height**	**Body mass**	**BMI percentile**	**Maturity offset**
**Gymnastics experience**	**–**					
**Age**	0.97	**–**				
**Body height**	0.74	0.76	**–**			
**Body mass**	0.83	0.81	0.90	**–**		
**BMI percentile**	ns	ns	ns	ns	**–**	
**Maturity offset**	0.91	0.94	0.89	0.92	ns	**–**
**ACROBATIC GYMNASTS (*n*** **= 10)**						
**Measure**	**Gymnastics experience**	**Age**	**Body height**	**Body mass**	**BMI percentile**	**Maturity offset**
**Gymnastics experience**	**–**					
**Age**	ns	**–**				
**Body height**	ns	ns	**–**			
**Body mass**	ns	ns	0.79	**–**		
**BMI percentile**	ns	ns	0.63	0.94	**–**	
**Maturity offset**	ns	0.91	0.65	ns	ns	**–**

^a^ *p* < 0.05

Maturity offset - years from age at peak height velocity, *ns* nonsignificant.

## Discussion

The aim of this study was to determine whether quiet standing postural sway (represented by the anterior-posterior and medial-lateral COP mean velocities under eyes-open and eyes-closed conditions) was different between young female artistic and acrobatic (carrying out the role of the “tops”) gymnasts. We also searched for correlations between the gymnasts’ postural sway velocities and their training experience, ages, body heights, body masses, BMI percentiles, and maturity offsets. The nonsignificant between-group differences observed in the postural sway velocities suggest similar postural steadiness in the artistic and acrobatic gymnasts under both visual conditions. However, it is worth mentioning that on average, artistic gymnasts swayed slower in the anterior-posterior direction under eyes-open conditions and the difference between the two groups (effect size) was medium. As expected, visual deprivation had an impact on the increase in postural sway velocities in both artistic and acrobatic gymnasts. In turn, as was hypothesized, the results of the correlations were not always analogous for the two groups.

The present study indicates that the longer training experience of young adolescent artistic gymnasts was strongly related to their lower anterior-posterior postural sway velocity (better postural steadiness) under eyes-open conditions. It is possible that the artistic gymnasts’ specific high-intensity training regimen [26, 27] contributed to such relationship. One of the goals in artistic gymnastics training is to achieve a perfectly steady bipedal posture (“stuck landing”) in the final phase of aerial routines. Among the most important muscle groups engaged in the “stuck landing” are foot plantar and dorsal flexors [35, 36]. Because these muscles also play the greatest role in anterior-posterior quiet standing postural control [6], it is possible that longer practicing and perfecting of the “stuck landing” also resulted in improved anterior-posterior postural steadiness. The relationship between the length of the training experience and the anterior-posterior postural sway velocity was only observed during trials with normal visual conditions, possibly because gymnasts practice their routines with their eyes open, and visual information is used to enhance the stability of the landing [36].

In turn, this study indicates that in the acrobatic gymnasts (practicing as “tops”) the length of their training experience was not reflected by specific changes in their quiet standing postural sway. Although the performance level of the young adolescent gymnasts in both groups was national, training experience in the acrobatic gymnasts was shorter (the mean length of training experience in the acrobatic gymnasts was 3.3 years and it was 4.8 for the artistic gymnasts). It is therefore possible that the training experience of the acrobatic gymnasts was not long enough to influence the characteristics of their quiet standing postural sway. Similarly, in 6–8-year-old children who practiced artistic gymnastics on average for 2.75 years, the duration of training experience was poorly related with static balance assessed with Stork Balance Stand test [28].

The shorter duration and lower frequency of the acrobatic gymnasts’ training sessions as well as their specific training regimen might have influenced the results as well. It may be considered that for the “top” acrobatic gymnast, it is not as difficult to maintain a steady bipedal posture during the final phase of the aerial routine (the “stuck landing”) as it is for the artistic gymnast because during this phase, the “top” gymnast is being safeguarded by the “base” gymnast (s). Perhaps practicing landing with the external support of the partner gymnast (s) also contributed to nonsignificant relation between the duration of the training experience and the postural sway in the acrobatic gymnasts.

The present study indicates that in young adolescent artistic gymnasts, not only a longer training experience, but also older age, greater body mass, body height, and biological maturity were strongly or very strongly related to better anterior-posterior postural steadiness. Since in this group the length of training experience, age, and anthropometric factors were also positively correlated with each other, it appears that the relationship between training experience and anterior-posterior steadiness in the artistic gymnasts went hand in hand with their maturation and physical development. Concomitantly, in the non-athletes a poor correlation between the anthropometric measures and postural steadiness was observed. This allows the authors to conclude with greater certainty that the result of the strong relationship between the artistic gymnasts’ training experience and their anterior-posterior steadiness was not coincidental.

Similar to the results obtained for the non-athletes, the age, body height, and maturity offset of the “top” acrobatic gymnasts were poorly related to their postural steadiness. Unlike the artistic gymnasts, the age of the acrobatic gymnasts was poorly correlated with their height, which suggests that older acrobatic gymnasts were not necessarily those who were taller. Although there were nonsignificant age differences between the gymnasts of the two disciplines, none of the “top” acrobatic gymnasts represented the age of 13 years. Concomitantly, the “top” acrobatic gymnasts were characterized by a lower body height (by 7 cm on average) compared to that of the artistic gymnasts, which suggests that having a low height was one of the strong determinants of their high (national level) performance. In the group of “top” acrobatic gymnasts, lower standard deviations from mean age, height, or maturity offset were also observed, indicating less intra-group variability. It is possible that too much intra-group similarity prevented the demonstration of a stronger association between these measures and postural sway velocity. However, this study indicates, as in the artistic gymnasts, that the greater body mass of the acrobatic gymnasts was strongly related to their better anterior-posterior postural steadiness. Interestingly, in the acrobatic gymnasts the association was observed regardless of the visual conditions. In the eyes-closed trials, greater body mass was also related to better medial-lateral steadiness. These results may suggest that when vision was not available, the acrobatic gymnasts who demonstrated greater body mass used their redundant system more effectively in the control of quiet standing. The above mentioned results are not in agreement with the investigation on dynamic stability in pre- and postpubertal female gymnasts practicing trampoline. That study demonstrated a poor correlation between gymnasts’ body height and mass and their dynamic body sway frequencies acquired while standing on a seesaw platform [3]. In another study of younger children (aged 6–8 years) practicing artistic gymnastics, static balance assessed with the simple balance test was negatively correlated with their body height and positively correlated with their body mass [28]. The comparison of the results of the present investigation with those studies and the interpretation of their discrepancies would be unjustified because the divergence in the sample characteristics and research methodology is too large.

In the present study, the acrobatic gymnasts’ higher BMI percentiles were also strongly related to better anterior-posterior postural steadiness under normal visual conditions and moderately related to better anterior-posterior and medial-lateral steadiness under visual deprivation conditions. Body mass index is a function of mass and height; however, the BMI percentiles for children are age- and sex-specific. Therefore, this study indicates that in some of the acrobatic gymnasts, not only greater body mass but also higher values of the body mass and square of the body height ratio specific to their age and sex were related to better overall postural steadiness. Interestingly, the artistic gymnasts’ BMI percentiles were poorly related to their postural steadiness. Based on these study results, the authors are not able to explain why artistic gymnasts and acrobatic gymnasts training as “tops” demonstrated different strengths of association between postural steadiness and body mass or BMI percentiles. Further research is necessary to investigate this phenomenon. Perhaps a deeper evaluation of postural sway variability and regularity (based on sample entropy assessment) or stabilogram decomposition into rambling and trembling components will make the interpretation of the strength of such correlations possible.

As it has been already mentioned, this study shows that the anthropometric measures of age-matched non-athletes were poorly related to their postural steadiness. These results are similar to the studies on larger non-athlete populations which examined 57 children age 7–18 [9] and 152 children age 7–12 [10]. According to these studies, the children’s postural stability was poorly correlated with their body mass and BMI, and it was poorly to weakly positively correlated with their body height. However, a weak to moderate positive correlation was observed between age and postural stability [9, 10]. This suggests that in order to detect the impact of age on postural sway characteristics in non-athlete children, a larger sample size with a larger age range is needed.

Although the young untrained females in the present study demonstrated age and biological maturity similar to that of the gymnasts, they were characterized by their greater body height, mass and BMI percentiles, and larger diversity of their anthropometric characteristics. It is possible that if the group is more diverse in regard to body proportions and composition (the percentage of muscle and fat tissue), the demonstration of the relationship between anthropometric measures and postural steadiness becomes impossible.

The results of this study should be considered with caution because of the relatively small sample size (the recruitment of larger number of 10–13-year-old, national level, female gymnasts in the Upper Silesian region was not possible). On the other hand, the strength of this study are rigorous inclusion and exclusion criteria resulting in the relatively large homogeneity of the selected groups of young adolescent female gymnasts in regard to their somatotype.

## Conclusions

The study suggests similar postural steadiness in the young adolescent artistic and acrobatic gymnasts; however, certain components of postural sway characteristics were related to discipline-specific training experience and anthropometric characteristics. Better anterior-posterior steadiness of the artistic gymnasts was associated with their longer training experience, greater age, body height, body mass, and biological maturity. Such relationships were observed only if vision was available for the gymnasts. In turn, the acrobatic gymnasts’ better anterior-posterior and medial-lateral steadiness was associated with their greater body mass and BMI percentiles under both normal and visual deprivation conditions. The non-athletes’ age and anthropometric measures were not related to their postural steadiness. In the assessment of quiet standing, the directional subcomponents of COP mean velocity turned out to be sensitive measures to detect discipline-specific relations between postural steadiness and training experience or anthropometric characteristics in young female gymnasts.

To our best knowledge, the present study is the first to compare postural steadiness between young gymnasts practicing two distinct disciplines as well as to investigate the association of their postural sway with their training experience and anthropometric measures.

The main results suggest that when training experience is long enough in young gymnasts, its relationship with COP measures acquired during simple quiet standing can be observed. Our results should be a foundation for future investigations which may lead to practical implications in the assessment of training results or in selecting children for certain gymnastic disciplines (considering their somatotype) with the use of posturography. Further studies are needed to determine whether reduced quiet standing postural sway velocity observed in young female gymnasts under laboratory conditions is related to their better landing control. Researchers should also focus on investigating correlations between the body building somatotype of young gymnasts (evaluated with more advanced objective methods, e.g., dual energy x-ray absorptiometry, anthropometry, or bioimpedance) and tasks which are discipline-specific. If the correlations exist, a child’s somatotype/anthropometric characteristics might become one of the factors considered when choosing a particular gymnastic discipline.

## Data Availability

Data analysed during this study are available from the corresponding author upon request.

## Abbreviations

COP: Centre of foot pressure
BMI: Body mass index
ES: Effect size
